# The effect of educational intervention based on social media on mental health literacy of high school students in Ramhormoz city: study protocol of a randomized controlled trial

**DOI:** 10.3389/fpsyg.2024.1377760

**Published:** 2024-12-20

**Authors:** Abouzar Nazari, Azadeh Askari, Abbas Rahimi Foroushani, Gholamreza Garmaroudi

**Affiliations:** ^1^Department of Health Education and Promotion, Faculty of Health, Tehran University of Medical Sciences, Tehran, Iran; ^2^Department of Psychology, Faculty of Psychology and Educational Sciences, Shahid Beheshti University, Tehran, Iran; ^3^Department of Epidemiology and Biostatistics, School of Public Health, Tehran University of Medical Sciences, Tehran, Iran; ^4^Department of Health Education and Promotion, School of Public Health, Tehran University of Medical Sciences, Tehran, Iran

**Keywords:** educational intervention, social media, mental health literacy (MHL), high school students, study protocol, randomized controlled trial, Iran

## Abstract

**Background:**

Adolescence is a critical period for developing and maintaining good habits for mental health and well-being. This is crucial for future mental health; as most mental health problems emerge during adolescence. Mental health literacy forms the foundation for preventing mental health issues, reducing stigma, and enhancing the effectiveness of help-seeking, particularly among adolescents.

**Objective:**

This study aims to measure the effect of social media-based educational intervention on mental health literacy among male high school students in Ramhormoz city.

**Methods:**

This randomized controlled trial (RCT) evaluated the effectiveness of a social media-based intervention, designed around the core components of *The Guide* training package. The study involves all high schools in Ramhormoz city using a stratified random sampling method. A total of 204 students are expected to participate. Measures of mental health literacy and attitudes towards seeking professional psychological help are evaluated at three time points: before the intervention (baseline), 3 months, and 6 months after the intervention.

**Results:**

We anticipate a significant improvement in the mental health literacy score of the intervention group compared to the control group at post-intervention and both follow-ups. Additionally, we expect a substantial enhancement in the attitude towards seeking professional psychological help score for the intervention group compared to the control group at post-intervention and 6-month follow-up, though not at the 3-month follow-up.

**Conclusion:**

In conclusion, this study will contribute valuable insights into the effectiveness of social media-based interventions in enhancing mental health literacy and attitudes towards seeking professional help among high school students. The results will guide future initiatives in mental health education and promotion.

**Clinical trials:**

This study protocol is registered with the Iranian Registry of Clinical Trials (IRCT) under the registration number IRCT20230603058372N1, dated June 5, 2023. The study adheres to the Standard Protocol Items: Recommendations for Interventional Trials (SPIRIT) guidelines, as outlined in Supplementary File S1.

## Introduction

Adolescence, the transformative phase from childhood to adulthood, is marked by unique challenges, prominently among them the increasing prevalence of mental health issues both globally and within Iran. International studies underscore a significant proportion of adolescents worldwide grappling with mental health challenges such as anxiety and depression. In Iran, a recent meta-analysis indicates that the prevalence of depression among adolescents ranges from 10 to 18%, while anxiety disorders affect up to 30% of this population (World Health Organization, 2020; Kuek et al., 2023; Hoseini-Esfidarjani et al., 2022; Heizomi et al., 2020).

The transition from childhood to adolescence, characterized by heightened vulnerability, societal pressures, and evolving interpersonal dynamics, contributes to the complexity of mental health issues in this demographic. Recognizing the urgency of addressing these concerns, our study endeavors to contribute to the broader discourse on adolescent mental health literacy (MHL) through innovative and contextually relevant interventions (Heizomi et al., 2020; Jafari et al., 2021; Jorm, 2012; Patton et al., 2016).

Mental health literacy (MHL), defined as the knowledge and beliefs enabling the recognition, management, and prevention of mental disorders (Jorm, 2012), emerges as a critical determinant of mental health outcomes. It influences help-seeking behavior, aids in stigma reduction, and facilitates early intervention (Kitchener and Jorm, 2002). However, MHL is often inadequate among young people, particularly those at high risk of developing mental health problems (Bröder et al., 2017).

While school-based interventions have proven effective in improving MHL among adolescents (Anderson et al., 2022; Cairns and Rossetto, 2019), traditional approaches face challenges such as limited resources, time constraints, and low engagement. Consequently, there is a need to explore innovative and alternative methods to deliver MHL education in schools (Lehtimaki et al., 2021; Zsila and Reyes, 2023).

Social media, widely used and preferred by young people, presents a potential medium to enhance MHL education. Offering interactive and personalized features, it enables peer-to-peer communication and support (Zsila and Reyes, 2023; Arshad et al., 2019). However, it also poses risks and challenges, including privacy, ethical, and quality issues, as well as the potential negative impact on mental health (Raeside et al., 2022). This underscores the need to design and evaluate evidence-based, ethical, and engaging social media-based interventions for MHL education.

Building on the recommendations of global health organizations (World Health Organization, 2018; American Psychological Association Washington, 2023), our study leverages the pervasive influence of social media to enhance MHL among high school students in Ramhormoz city. The study protocol outlines a randomized controlled trial (RCT) designed to assess the effectiveness of an educational intervention delivered through social media in addressing specific mental health challenges faced by teenagers in this region.

The mental health care system for children and adolescents in Iran faces significant challenges, with a shortage of qualified professionals and unmet needs. Iran has less than 0.3 child psychiatrists per 100,000 children, reflecting a substantial gap in service provision, particularly in underserved areas (Zarafshan et al., 2021; Sharifi et al., 2016). Studies show that a high percentage of Iranian adolescents experience mental health issues, yet mental health care remains inadequate, particularly in lower-income regions (Sharifi et al., 2016). Furthermore, while there are efforts, such as UNICEF’s programs, to improve child mental health services, these interventions have limited reach and effectiveness in addressing the widespread need for mental health care (Zarafshan et al., 2021; Sharifi et al., 2016).

Ramhormoz, situated in the southwestern province of Khuzestan, Iran, serves as the focal point for our investigation. With a population of approximately 127,000 people primarily engaged in agriculture, the city retains a close-knit community feel, fostering interconnected relationships among its residents. While the penetration rate of social media is not known, it’s important to consider that owning a smartphone is a common criterion for participation in our study. Our deliberate choice of Ramhormoz as the study setting aims to unravel the nuanced interplay between traditional values, economic constraints, and the mental health landscape of its adolescents. By amalgamating insights from global prevalence trends, the unique socio-cultural context of Iran, and the evolving discourse on mental health literacy (MHL), our research aspires to contribute to the development of impactful strategies for promoting the mental well-being of adolescents in Ramhormoz city and beyond.

## Method/design

**Figure 1:**
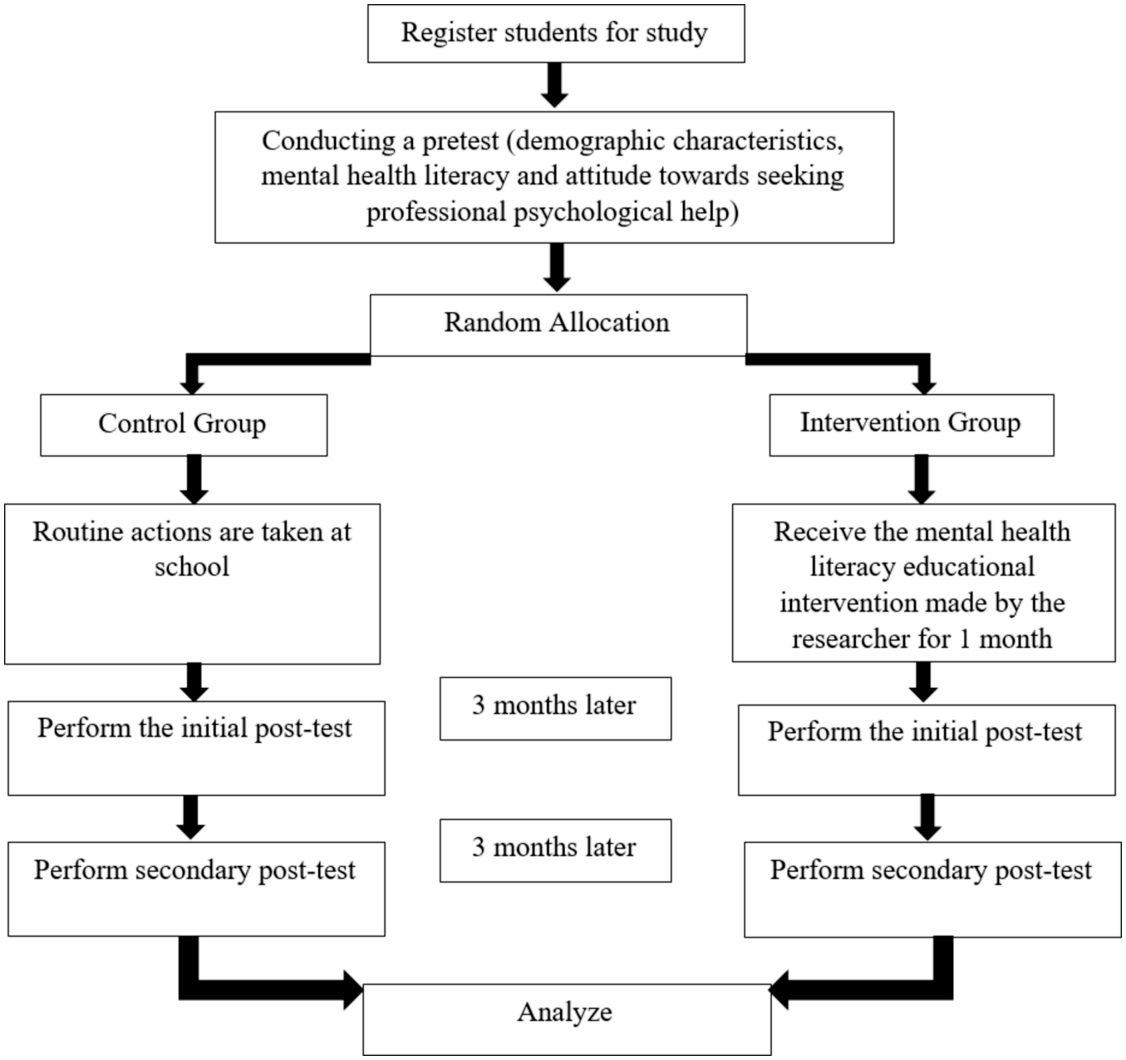
CONSORT flow diagram illustrating participant allocation and progress through the trial.

We used a CONSORT statement to describe the study – see Figure 1. The study design was a randomized controlled trial(RCT) with pre-test, post-test and control group without blinding. The participants will high school students from Ramhormoz city (Iran).

### Sampling method

This study uses a stratified random sampling method that focuses exclusively on male participants aged 14–20 years. First, a school district is randomly selected from among the available districts. Within the selected district, schools are stratified and then randomly assigned to intervention and control groups. Three schools are assigned to the control group and three schools to the intervention group. A total of 102 male students will be randomly selected from all three schools in each group. The number of participants selected from each school will be proportional to the student population of that school, ensuring fair representation.

Male students were selected for this study due to specific cultural, religious, and logistical constraints. In the study setting, adolescent male students are the primary population with regular and independent access to mobile phones, a key requirement for participation in this digital communication-based intervention. Additionally, the male researcher’s access to female students’ mobile phone numbers is restricted by cultural norms and policies imposed by the Ministry of Education and Culture. These factors make the inclusion of female participants impractical within the framework of this study and necessitate a focus on male students to ensure the feasibility and integrity of the research design.

### Sample size calculation

**Figure 2:**
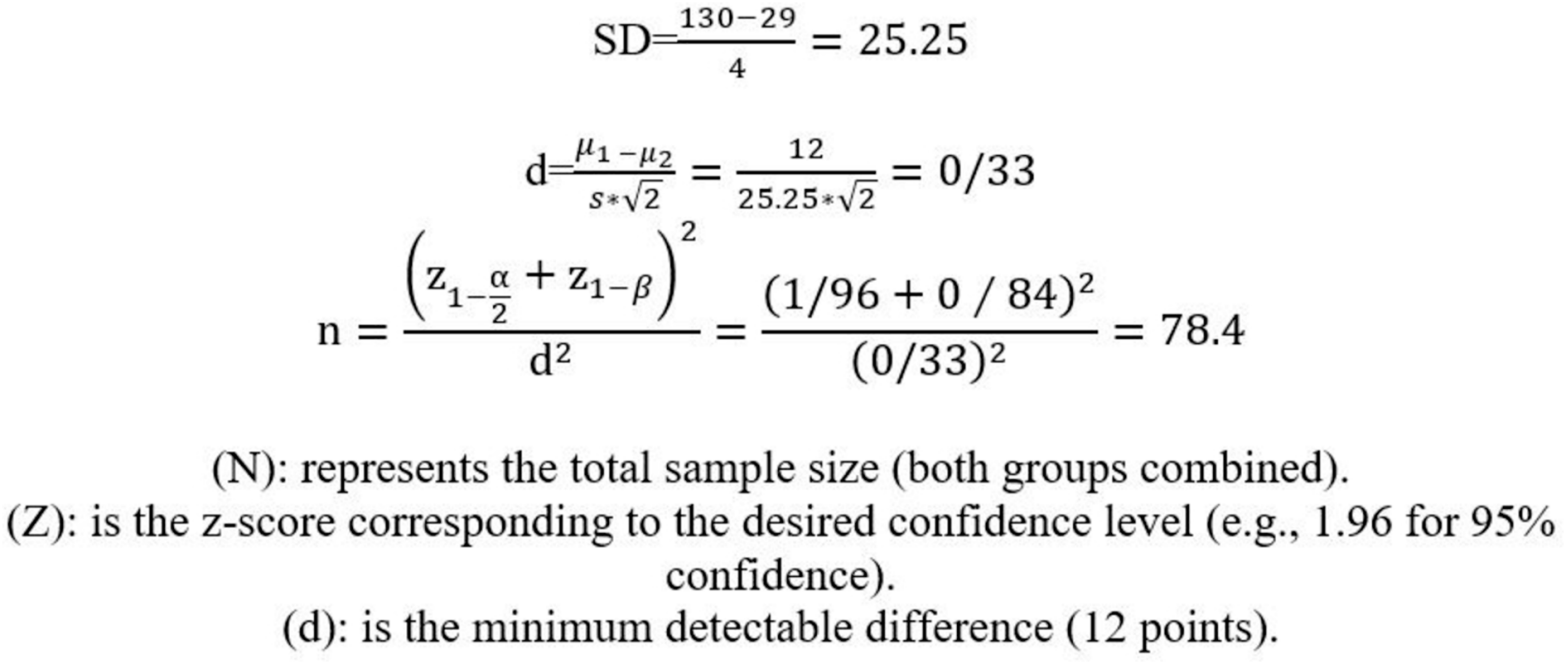
Sample size formula.

The main goal of this study is to determine the effect of education on the average score of mental health literacy in two intervention and control groups, which will be analyzed as the difference between the two averages. Since the minimum and maximum score of mental health literacy is between 29 and 130, therefore, the standard deviation of 25.25 points was considered and the number of samples in each group was determined in such a way that with 95% confidence and 80% test power, if the difference between the two averages A score greater than 12 is statistically significant. From the following formulas, the number of participants in each group was stated (Figure 2).

Therefore, for each of the intervention and control groups, 102 students will be randomly selected from among the second-high school students of Ramhormoz city (including 30% chance of dropping out). We try to select the number of people in each grade equally.

### Ethics approval and consent to participate

#### Recruitment and consent

Ethical approval for the study was granted by the Ethics Committee of Tehran University of Medical Sciences (Approval date: 27/05/2023, Reference: IR.TUMS.SPH.REC.1402.075). This study aimed to assess the impact of a social media-based educational intervention on mental health literacy. Both participants and their parents were informed that participation was entirely voluntary, their data would remain confidential, and findings would only be presented in aggregate form for research purposes.

#### Consent and assent collection process

##### To ensure ethical rigor and avoid potential coercion

- *Parental Consent:* Consent was obtained electronically through an online form sent via parents’ mobile numbers provided by the school. Parents reviewed the study details and acknowledged their consent by clicking “agree.”
- *Adolescent Assent:* After parental consent was granted, adolescents were presented with a separate assent form within the online interface. They were individually informed about the study’s objectives, procedures, and voluntary nature in a private virtual setting. Adolescents had the opportunity to review and decide independently whether to participate by selecting “agree.”

#### Informed consent and protections

Detailed information about the study objectives, expected procedures, potential risks and benefits, confidentiality measures, and the voluntary nature of participation was provided to both participants and their parents or guardians.

#### Withdrawal option

Both participants and their parents were informed that they could withdraw from the study at any time without any penalties or negative consequences.

This dual-step consent and assent process was designed to uphold ethical standards and ensure that both parents and adolescents made fully informed, independent decisions.

#### Data protection

To ensure participant privacy and confidentiality, all collected data will be anonymized and securely stored.

#### Compensation for human subjects research

As a token of appreciation for their time and participation, participants will receive compensation in the form of a 10 GB internet package for 1 month.

#### Dissemination of results

Study results will be shared with the scientific community through publications and presentations, contributing to the broader understanding of mental health literacy interventions.

#### Inclusion/exclusion criteria

##### Inclusion criteria

To be eligible for this study, male students enrolled in public high schools in Ramhormoz city must meet the following criteria:

- Have access to a smartphone with an internet connection.
- Demonstrate an understanding of both written and spoken Persian language.
- Express willingness to participate in the research and provide informed consent.
- Not having any known mental disorders or depressive symptoms.

#### Exclusion criteria

Participants will be excluded from the analysis if they:

- Withdraw their consent or express unwillingness to continue the research at any stage.
- Migrate to another city or school during the study period.

#### Intervention

Our planned intervention will utilize the Iranian social media platforms, Rubika and Eitaa, to disseminate the educational content of The Guide. The first author, acting as the primary facilitator, will conduct the program, which will be delivered through both synchronous and asynchronous formats. This blended approach allows for flexibility in learning while maintaining key engagement opportunities.

##### Facilitator and coordination

The first author will lead the educational program, organizing briefing sessions to ensure participants are well-informed about the program’s objectives, procedures, and expectations. These sessions will be conducted synchronously, allowing real-time interaction for better understanding and coordination.

##### Implementation details

The program will be structured around The Guide, with a thematic framework (outlined in Table 1) guiding the content. Methods will be in place to facilitate deep engagement, including real-time sessions and asynchronous materials for flexible access.

**Table 1:** Thematic framework of mental health literacy and methods of deepening and feedback.

	The subject of intervention	Deepening and feedback
1	Introduction: Mental health, mental disorders and brain functions	Question and Answer
2	The stigma Definition of stigma Stigma: Myths and realities of mental illness Which famous people lived with a mental illness?	Educational video Questions and Answers Stories
3	Information about specific mental illnesses What happens when the brain gets sick? Common mental illnesses Understanding common mental disorders in adolescents Sharing knowledge	Educational video Questions and Answers
4	Mental disorders of cognition and perception: psychotic disorders: Schizophrenia	Educational video Questions and Answers
5	Mental disorders of emotion and feeling: Mood disorders: Depression and bipolar disorder	Educational video Questions and Answers Stories
6	Mental disorder in signaling: Generalized anxiety disorder, social anxiety disorder and panic disorder	Educational video Questions and Answers
7	Other mental disorders with hyperarousal symptoms: Obsessive Compulsive Disorder and Post Traumatic Stress Disorder	Educational video Questions and Answers
8	Psychosomatic disorder: eating disorders Anorexia nervosa and bulimia nervosa	Educational video Questions and Answers
9	Behavioral disorders: ADHD, substance-related disorders, conduct disorder	Educational video Questions and Answers
10	Behaviors related to mental disorders: Suicide and self-harm	Educational video Questions and Answers Stories
11	Experiences of mental illness and the importance of family communication Seek help and find support	Question and Answer
12	Seek help and find support	Question and Answer
13	What if (future scenarios in case of certain symptoms)	Question and Answer
14	The importance of positive mental health	Educational video Questions and Answers
15	List of community mental health resources	Question and Answer

##### Duration and social media platforms

The intervention will span 1 month, consisting of 15 sessions. Each session will last approximately 30 min to 1 h, with 3–4 sessions delivered per week to ensure timely completion. The content will be regularly updated on Rubika and Eitaa, allowing participants to access both synchronous and asynchronous materials.

##### Incentives and participant engagement

Participants will be encouraged to actively engage through ongoing support, educational videos, and interactive Q&A sessions. Although the program is structured, participants will not have the flexibility to choose specific sessions based on preference, as all sessions are integral to the overall intervention. These strategies aim to maintain engagement throughout the study, with particular focus on improving retention, especially in the control group.

The educational content that the control participants receive at the end of the study will be provided in-person as a guide book rather than the online platform session content.

Please refer to Figure 1 for a visual representation of the intervention components, illustrating the topics covered and the methods employed for deepening and feedback.

### Data collection

In this randomized trial study, the statistical population consists of secondary school students from schools in Ramhormoz city. After coordinating with the schools and obtaining the phone numbers of the students, the link of the questionnaire that was created through Porsline website will be shared with them. The students will be asked to complete the study tool before, 3, and 6 months after the intervention.

### Primary outcome measure

#### Mental health literacy scale (MHLS)

This questionnaire was developed and validated by O’Connor in 2015. This scale has 35 items and 6 dimensions to measure the level of MHL. The Persian version of this questionnaire has 29 items that have been psychometrically evaluated by Nejatian et al. (2021), and it includes the following six sections:

- People’s ability to diagnose mental disorders: This feature consists of eight items (for example: Do you think personality disorder is considered other than mental illness). This feature is measured using a 4-point Likert scale (very unlikely, unlikely, likely, very likely) is measured with a score range of 8–32.
- Attitudes that promote appropriate help-seeking cognition or behavior: This trait includes 10 items (e.g.: If I have a mental disorder, I do not like to tell anyone). This trait is assessed using a 5-point Likert scale [(strongly disagree, disagree, neither agree nor disagree, agree, strongly agree) or (definitely willing, probably willing, neither willing nor unwilling, probably not willing, definitely not willing)] will be measured. Score range from 10 to 50.
- Awareness of self-healing: This feature includes two items (for example: if a person has a lot of difficulty managing his emotions, such as being anxious or depressed, how much do you think improving the quality of sleep would be useful for him?). This feature is measured using a 4-point Likert scale (not very useful, not useful, useful, very useful) with a score range of 2–8.
- Awareness of available professional help: This feature includes three items (e.g.: In your opinion, it is likely that cognitive behavioral therapy based on challenging negative thoughts and increasing beneficial behaviors). This feature is measured using a 4-point Likert scale (very unlikely, unlikely, likely, very likely) with a score range of 3–12.
- Awareness of where to look for information: This feature includes four items (e.g., I am sure that I can use the computer and telephone to look for information about mental disorders). This trait is measured using a 5-point Likert scale (Strongly Disagree, Disagree, Neither Agree nor Disagree, Agree, Strongly Agree) with a score range of 4–20.
- Awareness of risk factors and causes: this characteristic consists of two items (for example: in general, to what extent do you think men in Iran may experience anxiety disorders more than women). This feature is measured using a 4-point Likert scale (very unlikely, unlikely, likely, very likely) with a score range of 2–8.

A high score for each attribute indicates a higher literacy rate for each attribute. Also, the total score of MHLS is obtained from the sum of the scores of all features. The lowest score is 29 and the highest is 130. Higher scores indicate more favorable MHL status. Based on the reliability results, MacDonald’s omega coefficient and Cronbach’s alpha coefficient for all MHLS features were 0.797 and 0.789, respectively.

### Secondary outcome measures

#### Scale of attitude towards seeking professional psychological help

ATSPPH-SF is a 10-item scale used to assess ATSPPH on a 4-point Likert scale, ranging from 0 to 3. The total scores range from 0 to 30, where a higher total score indicates a more positive ATSPPH and is associated with lower levels of stigma against mental illness. This scale included two dimensions: (1) openness to seeking professional help for emotional problems (items 1, 3, 5, 6, 7) with item scores from zero (disagree) to three (agree) (2) value and need in seeking help professional (items 2, 4, 8, 9, 10) with reverse scoring of items (zero = agree and three = disagree).

### Statistical analysis

The statistical analysis of this study will be based on the intention-to-treat (ITT) principle, which includes all randomized participants regardless of their adherence to the intervention or their status at the end of the trial. The ITT analysis will preserve the balance of baseline characteristics achieved by randomization and reduce the risk of bias due to selective reporting or loss to follow-up (Hemming et al., 2020).

#### Potential confounds

Researchers anticipate several potential confounds that may influence the outcomes, such as:

1. Pre-existing Mental Health Conditions: Variability in participants’ baseline mental health status may affect their response to the intervention.
2. Socioeconomic Factors: Differences in socioeconomic status might influence participants’ access to mental health resources, potentially impacting outcomes.

The primary outcome of this study is the change in mental health literacy score (MHLS) from baseline to post-intervention and follow-up. The secondary outcome is the change in attitude towards seeking professional psychological help score from baseline to post-intervention and follow-up. Both outcomes will be measured using validated scales.

Demographic and baseline characteristics of the participants will be summarized using descriptive statistics, including mean, standard deviation, median, interquartile range, frequency, and percentage, as appropriate. The normality of the outcome variables will be assessed using graphical methods (histograms and Q-Q plots) and numerical tests (Shapiro–Wilk test). If the outcome variables are normally distributed, parametric tests will be employed; otherwise, non-parametric tests will be utilized.

Between-group differences in outcome variables at post-intervention and follow-up will be analyzed using analysis of covariance (ANCOVA), adjusting for baseline values and other potential confounders. Within-group changes in outcome variables from baseline to post-intervention and follow-up will be analyzed using paired t-tests or Wilcoxon signed-rank tests, depending on the normality assumption. Effect sizes and 95% confidence intervals will be reported for all comparisons.

We will employ techniques such as multilevel modeling (also known as hierarchical linear modeling) or generalized estimating equations (GEE) to model dependencies among observations within the same cluster. The level of significance for all statistical tests will be set at 0.05 (two-sided). All analyses will be conducted using SPSS software version 26.0.

## Results

### Baseline measures

Before delving into the results, it’s crucial to highlight that these findings are anticipated and based on assumptions, as the study has not been implemented yet.

### Participants’ baseline assessment

At this stage, participants will undergo assessments encompassing mental health literacy (MHL) scores and attitudes towards seeking professional psychological help. Encouragingly, the initial assumptions suggest no notable distinctions between the intervention and control groups at baseline, establishing a solid foundation for future comparisons.

### Anticipated adherence to the intervention

Assuming the successful implementation of the educational intervention, it is expected that participant adherence will be notably high within the intervention group. A majority of participants in this group are anticipated to complete the program, indicating a promising level of engagement and commitment.

### Primary outcome: mental health literacy (MHL)

In accordance with our assumptions, the intervention group is expected to display statistically significant advancements in MHL scores compared to the control group at each assessment juncture (*p* < 0.05). This suggests an anticipated sustained enhancement in MHL among those exposed to the intervention.

### Secondary outcome: attitude toward seeking professional psychological help

The intervention group, as per our hypothetical scenario, is expected to demonstrate a noteworthy improvement compared to the control group at the post-intervention and 3-month follow-up evaluations (*p* < 0.05). However, it is essential to note that, in our assumptions, this positive effect is not expected to endure at the 6-month follow-up. This suggests an anticipated potential necessity for ongoing support mechanisms or booster sessions to uphold the positive shift in attitudes over the long term.

### Subgroup analyses

While recognizing the anticipated nature of these findings, subgroup analyses will be tentatively explored to uncover potential variations in outcomes based on demographic factors such as gender, age, and socioeconomic status. Although overall improvements are expected, further investigation will be warranted once the study is executed to draw more concrete conclusions from these subgroup analyses.

## Discussion

The primary objective of this study is to assess the impact of an educational intervention based on social media on the mental health literacy of high school students in Ramhormoz city. Mental health literacy, defined as knowledge and beliefs aiding in the recognition, management, or prevention of mental disorders, encompasses various components crucial for fostering good mental health and addressing mental disorders (Jorm, 2012).

Social media, being a widely used and accessible platform for communication and information sharing among young people, holds the potential to enhance mental health literacy. However, the utilization of social media for mental health education brings forth challenges and risks, including exposure to misinformation, cyberbullying, and privacy concerns (Kutcher et al., 2016). Consequently, the study emphasizes the need for tailored and evidence-based educational interventions on social media, designed to meet the specific needs and preferences of the target audience (Kutcher et al., 2016).

The intervention in this study involves a series of culturally appropriate and interactive social media posts covering a range of mental health topics. The randomized controlled trial design, comparing an intervention group to a control group, will measure mental health literacy and attitudes toward seeking professional psychological help as primary and secondary outcomes, respectively.

### Strengths of the study

1. *Researcher-Developed Intervention:* The study leverages a researcher-developed intervention based on the core components of the Mental Health and High School Curriculum Guide. This enhances the attractiveness and facilitates accurate comparisons with other studies utilizing the Guide.
2. *Use of Social Media Platform:* Instead of traditional face-to-face training, the study capitalizes on the popularity of social media, particularly among students and adolescents. This approach aims to create a more engaging and flexible intervention aligned with students’ free time.
3. *Two Three-Month Follow-Up Phases:* The study’s inclusion of two three-month follow-up phases strengthens its design by assessing the maintenance of intervention results over time.

### Challenges and limitations

1. *School Cooperation:* Anticipated challenges include potential non-cooperation from schools. Efforts will be focused on engaging education officials and teachers to support study referrals and enhance student engagement, rather than relying solely on lobbying and advocacy.
2. *Student Non-Cooperation:* Non-cooperation from some students is another potential limitation. Designing educational content in an attractive way aims to mitigate this factor.
3. *Short Follow-Up Period*: The study acknowledges the relatively short follow-up period of approximately 6 months. The hope is to demonstrate significant benefits within this timeframe, encouraging the development of mental health literacy programs.

## Conclusion

In conclusion, this study seeks to evaluate the effectiveness of a social media-based educational intervention on the mental health literacy of high school students in Ramhormoz city. Anticipating significant improvement in mental health literacy and attitudes toward seeking professional psychological help in the intervention group, the study aims to demonstrate the efficacy of using social media for mental health education. The expected positive reception from participants indicates increased knowledge, awareness, and confidence in dealing with mental health issues.

The study’s outcomes are poised to offer valuable insights for policymakers, educators, health professionals, and researchers interested in utilizing social media for mental health promotion. By showcasing the effectiveness of social media as a platform for mental health education in a resource-constrained setting, the study provides evidence-based and culturally tailored strategies. The implications extend beyond Iran, influencing the development of programs that foster positive attitudes toward mental health among adolescents in similar contexts.

Acknowledging potential challenges and limitations, the study encourages further research to address these constraints and explore sustained positive effects over an extended period.

### Trial status

This trial is still in the planning stage and has not been implemented yet. The trial protocol has been approved by the Ethics Committee of Tehran University of Medical Sciences (IR.TUMS.SPH.REC.1402.075). The trial has been registered in the Iran Randomized Clinical Trial Center (IRCT20230603058372N1 on June 5, 2023). The recruitment of participants is expected to start in June 2023 and end in July 2023. The intervention phase is expected to start in August 2023 and end in September 2023. The follow-up phase is expected to start in November 2023 and end in January 2024. The data analysis and manuscript writing are expected to start in February 2024 and end in March 2024. The anticipated date of completion of the trial is April 2024.

## References

[ref1] American Psychological Association Washington (2023). Health advisory on social media use in adolescence. DC: American Psychological Association Washington.

[ref2] Anderson S. Schurig M. Sommerhoff D. Gebhardt M. (2022). Students’ learning growth in mental addition and subtraction: results from a learning progress monitoring approach. Front. Psychol. 13:944702. doi: 10.3389/fpsyg.2022.944702, PMID: 36518966 PMC9742475

[ref3] Arshad A. Ahmad Hanan M. Saleem N. Farzooq S. Fatima R. (2019). Media and mental health literacy: do mediated interventions enhance mental health awareness? Implications and policy recommendations. Int. J. Ment. Health Promot. 21, 99–109. doi: 10.32604/IJMHP.2019.010834

[ref4] Bröder J. Okan O. Bauer U. Bruland D. Schlupp S. Bollweg T. M. . (2017). Health literacy in childhood and youth: a systematic review of definitions and models. BMC Public Health 17:361. doi: 10.1186/s12889-017-4267-y, PMID: 28441934 PMC5405535

[ref5] Cairns K. Rossetto A. (2019). School-based mental health literacy interventions. Literacy, 291–306. doi: 10.56687/9781447344520-022

[ref6] Committee on Publication Ethics (2000). Committee on publication ethics: the COPE report 1999. Guidelines on good publication practice. Occup. Environ. Med. 57, 506–509. doi: 10.1136/oem.57.8.506, PMID: 10896956 PMC1740000

[ref7] Heizomi H. Allahverdipour H. Jafarabadi M. A. Bhalla D. Nadrian H. (2020). Effects of a mental health promotion intervention on mental health of Iranian female adolescents: a school-based study. Child Adolesc. Psychiatry Ment. Health 14, 1–10. doi: 10.1186/s13034-020-00342-6 32983257 PMC7510259

[ref8] Hemming K. Kearney A. Gamble C. Li T. Jüni P. Chan A. W. . (2020). Prospective reporting of statistical analysis plans for randomised controlled trials. Trials 21, 1–2. doi: 10.1186/s13063-020-04828-8, PMID: 33115516 PMC7594284

[ref9] Hoseini-Esfidarjani S.-S. Tanha K. Negarandeh R. (2022). Satisfaction with life, depression, anxiety, and stress among adolescent girls in Tehran: a cross sectional study. BMC Psychiatry 22, 109–106. doi: 10.1186/s12888-022-03757-x, PMID: 35148694 PMC8840633

[ref10] Jafari A. Nejatian M. Momeniyan V. Barsalani F. R. Tehrani H. (2021). Mental health literacy and quality of life in Iran: a cross-sectional study. BMC Psychiatry 21, 1–11. doi: 10.1186/s12888-021-03507-5 34641793 PMC8507341

[ref11] Jorm A. F. (2012). Mental health literacy: empowering the community to take action for better mental health. Am. Psychol. 67, 231–243. doi: 10.1037/a0025957, PMID: 22040221

[ref12] Kitchener B. A. Jorm A. F. (2002). Mental health first aid training for the public: evaluation of effects on knowledge, attitudes and helping behavior. BMC Psychiatry 2, 1–6. doi: 10.1186/1471-244X-2-10 12359045 PMC130043

[ref13] Kuek J. H. L. Raeburn T. Liang A. G. Wand T. (2023). Mental health professionals' perspectives regarding how recovery is conceptualized in Singapore: a constructivist grounded theory study. J. Ment. Health 32, 736–743. doi: 10.1080/09638237.2023.2182431, PMID: 36866589

[ref14] Kutcher S. Wei Y. Coniglio C. (2016). Mental health literacy: past, present, and future. Can. J. Psychiatry 61, 154–158. doi: 10.1177/0706743715616609, PMID: 27254090 PMC4813415

[ref15] Lehtimaki S. Martic J. Wahl B. Foster K. T. Schwalbe N. (2021). Evidence on digital mental health interventions for adolescents and young people: systematic overview. JMIR Ment. Health 8:e25847. doi: 10.2196/25847, PMID: 33913817 PMC8120421

[ref16] Leung T. I. de Azevedo Cardoso T. Mavragani A. Eysenbach G. (2023). Best practices for using AI tools as an author, peer reviewer, or editor. J. Med. Internet Res. 25:e51584. doi: 10.2196/51584, PMID: 37651164 PMC10502596

[ref17] Nejatian M. Tehrani H. Momeniyan V. Jafari A. (2021). A modified version of the mental health literacy scale (MHLS) in Iranian people. BMC Psychiatry 21, 53–11. doi: 10.1186/s12888-021-03050-3, PMID: 33485306 PMC7824912

[ref18] Patton G. C. Sawyer S. M. Santelli J. S. Ross D. A. Afifi R. Allen N. B. . (2016). Our future: a lancet commission on adolescent health and wellbeing. Lancet 387, 2423–2478. doi: 10.1016/S0140-6736(16)00579-1, PMID: 27174304 PMC5832967

[ref19] Raeside R. Spielman K. Maguire S. Mihrshahi S. Steinbeck K. Kang M. . (2022). A healthy lifestyle text message intervention for adolescents: protocol for the Health4Me randomized controlled trial. BMC Public Health 22:1805. doi: 10.1186/s12889-022-14183-9, PMID: 36138375 PMC9503214

[ref20] Sharifi V. Mojtabai R. Shahrivar Z. Alaghband-Rad J. Zarafshan H. Wissow L. (2016). Child and adolescent mental health Care in Iran: current status and future directions. Arch. Iran. Med. 19, 797–804, PMID: 27845550

[ref21] World Health Organization. (2018). Health for the world's adolescents: A second chance in the second decade. Available at: https://www.who.int/publications-detail-redirect/WHO-FWC-MCA-14.05 (Accessed January 1, 2014).

[ref22] World Health Organization. (2020). Adolescent mental health. Available at: https://www.who.int/news-room/fact-sheets/detail/adolescent-mental-health (Accessed October 10, 2024).

[ref23] Zarafshan H. Wissow L. S. Shahrivar Z. Mojtabai R. Khademi M. JafariNia M. . (2021). Children and adolescents' mental health in Iran's primary care: perspectives of general practitioners, school staff and help seekers. Glob. Soc. Welf. 8, 1–10. doi: 10.1007/s40609-019-00144-5, PMID: 33738179 PMC7962553

[ref24] Zsila Á. Reyes M. E. S. (2023). Pros & cons: impacts of social media on mental health. BMC Psychol. 11:201. doi: 10.1186/s40359-023-01243-x, PMID: 37415227 PMC10327389

