# Supplementary figures and images for: The effect of educational intervention based on social media on mental health literacy of high school students in Ramhormoz city: study protocol of a randomized controlled trial
Source: Front Psychol. 2024 Dec 20;15:1377760. doi: 10.3389/fpsyg.2024.1377760 (PMC11695117; doi:10.3389/fpsyg.2024.1377760)

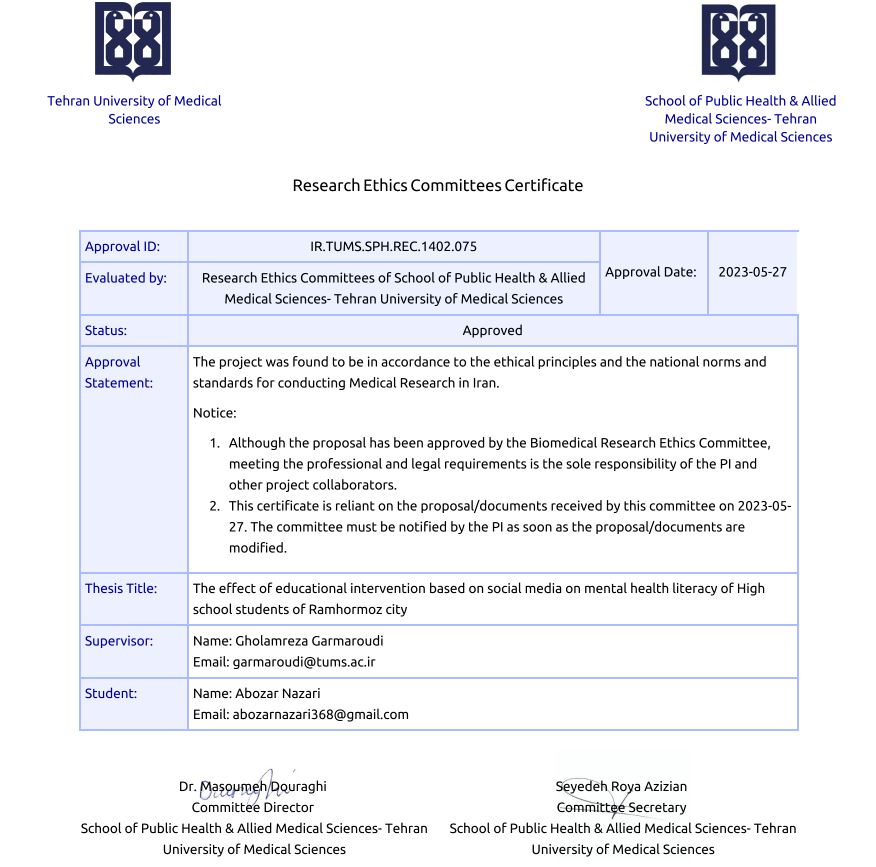

Supplement: Supplementary file 1 [file Image_1.jpg]
